# Comprehensive relationship between disease activity indices, mTSS, and mHAQ and physical function evaluation and QOL in females with rheumatoid arthritis

**DOI:** 10.1038/s41598-023-49380-y

**Published:** 2023-12-11

**Authors:** Tetsuyuki Nagafusa, Takashi Mizushima, Motohiro Suzuki, Katsuya Yamauchi

**Affiliations:** 1 Department of Rehabilitation Medicine, Hamamatsu University School of Medicine, 1-20-1, Handayama, Higashi-Ku, Hamamatsu, Shizuoka 431-3192 Japan; 2 Department of Rehabilitation Medicine, Dokkyo Medical University, Mibu, Shimotsuga, Tochigi Japan; 3 Department of Orthopaedic Surgery, Omaezaki City General Hospital, Omaezaki, Shizuoka Japan; 4 Department of Orthopaedic Surgery, Hamamatsu University School of Medicine, Hamamatsu, Shizuoka Japan

**Keywords:** Rheumatoid arthritis, Quality of life, Health care, Rheumatology

## Abstract

Rheumatoid arthritis (RA) causes significant physical disability. We comprehensively investigated the relationship between RA disease activity (Disease Activity Score 28-C-reactive protein [DAS28-CRP], Simplified Disease Activity Index [SDAI], and Clinical Disease Activity Index [CDAI]), physical function (10-Meter Walk Test [10 MWT], Timed Up and Go test [TUG], Functional Reach Test [FRT], and Disabilities of the Arm, Shoulder, and Hand [DASH]), and quality of life (QOL) (Short-Form 36 [SF-36®]). We also investigated the relationship between van der Heijde’s modified Total Sharp Score (mTSS), modified Health Assessment Questionnaire (mHAQ), and physical function and QOL assessments. Among 35 female patients with RA, DAS28-CRP correlated solely with DASH (*r* = 0.376), while SDAI and CDAI did not correlate with physical function. The mTSS-hand roentgenographic evaluation correlated with TUG (*r* = 0.359), FRT (*r* = − 0.415), and DASH (*r* = 0.533) among physical function assessments. The mHAQ correlated with 10 MWT (*r* = 0.347), TUG (*r* = 0.356), FRT (*r* = − 0.420), and DASH (*r* = 0.646). DAS28-CRP correlated with six of the eight subscales of SF-36®, and mTSS and mHAQ correlated with only one subscale. RA disease activity assessments may not reflect all physical functions and QOL domains of female patients with RA. Evaluating physical function and QOL in female patients with RA is essential.

## Introduction

Rheumatoid arthritis (RA) is a chronic autoimmune disease that causes synovial inflammation and joint erosion, leading to progressive functional disability^1^. In recent years, advances in disease-modifying anti-rheumatoid drugs (DMARDs) and tightly controlled treatment strategies (treat-to-target [T2T]) have improved the health status of patients with RA^2^. Current treatment goals are expressed using the T2T concept, which includes clinical remission followed by long-term prevention of joint destruction and preservation of physical function^3^. To achieve these goals, disease activity indices such as the Disease Activity Score (DAS) 28-C-reactive protein (CRP) [DAS28-CRP]^4–6^ and roentgenographic joint destruction assessment such as the van der Heijde's modified total sharpness score (van der Heijde's-mTSS)^7,8^ are often used when considering treatment strategies using drug therapy. Recently, simple activities of daily living (ADL) assessments such as the modified Health Assessment Questionnaire (mHAQ) have also been used^9^. However, although physical function and quality of life (QOL) are often evaluated in research, they are not commonly used to determine treatment strategy. Additionally, it is unclear whether ADL is sufficiently evaluated. Therefore, it is likely that rehabilitation treatment is not appropriately provided to patients with RA-related decreased physical function.

Recently, cross-sectional studies have been conducted on the relationship between RA disease activity, pain sensitisation, and physical function evaluation^10^. There are also studies on the relationship between disease activity and upper extremity function^11,12^, and the relationship between disease activity and lower extremity function^13^. However, these studies have been conducted separately, and to the best of our knowledge, there have been no studies that have comprehensively investigated the relationship between disease activity/roentgenographic joint destruction assessment of RA and physical function, ADL and QOL evaluations.

Our study aimed to investigate the relationship between RA disease activity/roentgenographic joint destruction/simple ADL assessment and physical function/detailed ADL/QOL assessments that are commonly used in rehabilitation treatment. Moreover, we aimed to clarify the importance of additionally assessing physical function and QOL, and to clarify the necessity of providing appropriate rehabilitation treatment.

## Methods

### Study design and ethics statement

We conducted a retrospective, single-centre study at Hamamatsu University Hospital. This study adhered to the tenets of the Declaration of Helsinki and was approved by the Institutional Review Board of Hamamatsu University School of Medicine (IRB approval no., 22-243). Patient consent was not required for this retrospective study.

### Participants

Based on medical record screening, we included patients with RA who visited the Department of Orthopaedic Surgery or Rehabilitation Medicine, Hamamatsu University School of Medicine, who received treatment between May 2012 and January 2013. The participation criteria was completion all of the following evaluations. The inclusion criteria were as follows: (1) completion of standard examinations and tests in RA practice, including the evaluation of joint findings (swelling and tenderness), blood examination of CRP, X-ray images of the hands and feet, and the mHAQ assessment; and (2) completion of examinations in rehabilitation therapy, including physical function measurement: 10-m Walk Test (10 MWT), Timed Up and Go Test (TUG), Functional Reach Test (FRT), and The Disability Arm, Shoulder, and Hand (DASH) test; and (3) completion of examinations including Functional Independence Measure (FIM) for assessing ADL and Short-Form 36 (SF-36®) for QOL assessment. All patients participating in the study underwent these assessments.

### RA disease activity assessment (DAS28-CRP, SDAI, and CDAI)

We measured the DAS 28-CRP, Simplified Disease Activity Index (SDAI), and Clinical Disease Activity Index (CDAI) to evaluate the disease activity in RA^4–6^. DAS 28-CRP enumerates the number of proximal interphalangeal joints (PIP), metacarpophalangeal joint (MCP), wrist, elbow, shoulder, knee, and 28 swollen and tender joints of the thumb to the little finger on both sides. Additionally, CRP and patients’ global assessment using the Visual Analog Scale (VAS) were calculated using specific formulas. The SDAI and CDAI are simplified methods of the DAS28^6^. The SDAI can be easily evaluated without any special formula and is calculated by adding the number of tender joints, the number of swollen joints, the patient's and physician's global assessment, and the CRP value. The CDAI further simplifies the SDAI and evaluates it without including blood test findings of CRP. The joint examination and measurements were performed by the first author.

### Roentgenographic joint destruction assessment in patients with RA (mTSS)

Joint destruction was evaluated using van der Heijde's-mTSS using radiography of both the hands and feet^7^. The van der Heijde’s mTSS was calculated by dividing the defined areas of the fingers, wrists, and toes on both sides into bone joint erosion (JE) (0–4 points) and joint space narrowing (JSN) (0–5 points) scores. Regarding the highest score per joint, the JE score was 5 for the fingers and wrists and 10 for the toes, whereas the JSN score for fingers, wrists, and toes was 4 points. The maximum total score is 448 points. Higher mTSS scores indicate more advanced joint destruction. Radiographic evaluation and measurements were performed by the first author.

### Activity of daily living assessment in patients with RA (mHAQ)

The mHAQ was measured as an ADL assessment^9^. The patients were asked questions and evaluated; the evaluation items comprised eight items related to dressing, standing, eating, walking, hygiene, movement, grip strength, and activity. The scoring was performed as follows: For each item that patients could perform easily by themselves: 0 points; for each item that patients could somewhat perform by themselves: 1 point; for each item that the patients could perform with someone else’s help: 2 points; and items that patients could not do at all: 3 points. The mHAQ-disability index (DI) was calculated by adding the points for the 8 items and dividing the value by 8.

### Physical function (10 MWT, TUG, FRT, and DASH), ADL (FIM), and QOL (SF-36®)) assessments used in rehabilitation

For physical function evaluation used in rehabilitation, the 10 MWT^14,15^, TUG^16–18^, and FRT^17,19^ were used to measure walking, dynamic balance, and static balance abilities, respectively. The 10 MWT assessed participants’ maximum walking speed over a short distance. A 10-m portion of the 14-m track was used^14,15^. In the 10 MWT, a larger value (seconds) means a slower walking speed. The TUG was used to assess dynamic balance function, in seconds; that is, the time an individual takes to stand up from a standard armchair, walk a distance of 3 m, turn, walk back to the chair, and sit down again. The participant did not use a customary walking aid (none, cane, or walker) in this study^16,18^. In the TUG, a larger value (seconds) means it takes longer to complete the task, indicating poor dynamic balance. The FRT assessed functional reach performance using the measuring device mounted on the wall at shoulder height. The subject was asked to reach as far forward as possible, in a plane parallel to the measuring device, without moving the support base^17,19^. In the FRT, smaller the values (centimetre) indicate worse the balances of the lower extremity. Upper extremity function was assessed using the Japanese version of DASH (DASH-JSSH)^20^. The DASH was a 30-item disability/symptom (DASH-DS) scale concerning the patient’s upper extremity. The items ask about the severity of each of the symptoms of pain, activity-related pain, tingling, weakness, and stiffness; the degree of difficulty when performing various physical activities because of an arm, shoulder, or hand problem; the effect of the upper extremity problem on social activities, work, and sleep; and the psychological effect on self-image. These provide DASH-DS score ranging from 0 (no disability) to 100 (most severe disability) after summation of the scores from all items and transformation^20^.

The FIM was used for the ADL assessment^21^. This measure consists of 13 mobility items and 5 communication and social cognition items. Participant ADL performance is assessed on a scale of 1–7. A full score is 126 points in total, meaning fully independent, and the lowest possible score is 18 points, meaning total assistance is required.

The SF-36v2 (Japanese version) was used for the QOL assessment^22^. This measure is a comprehensive health-related QOL scale and is commonly used for assessment with subjective outcome measures. The subscales consist of 8 sections: Physical Function (PF), Role Physical (RP), Body Pain (BP), General Health (GH), Vitality (VT), Social Function (SF), Role Emotional (RE), and Mental Health (MH). There are 36 questions. The raw score for each subscale can be from 0 to 100 points. Higher scores indicate higher QOL.

Two physical therapists assessed the 10 MWT, TUG, FRT, and FIM scores, whereas the DASH and SF-36® were self-administered questionnaires.

### Statistical analysis

Statistical analysis was performed using IBM SPSS software ver. 28.0 (IBM Corp., USA). We conducted a correlation analysis between DAS28-CRP/SDAI/CDAI and 10 MWT/TUG/FRT/DASH/FIM, respectively. Correlation analysis was also conducted between mTSS-hand/mTSS-foot/mTSS-total/mHAQ and 10 MWT/TUG/FRT/DASH/FIM, respectively. For analysis of SF-36®, we conducted a correlation analysis between DAS28-CRP/SDAI/CDAI/mTSS-hand/mTSS-foot/mTSS-total/mHAQ and RF/RP/BP/GH/VT/SF/RE/MH with subscales of SF-36®, respectively. The normality of the data was assessed using the Kolmogorov–Smirnov test. Our data were generally not normally distributed. Therefore, Spearman's rank correlation coefficient test was used as a non-parametric test for the statistical analysis, and the significance level was set at *p* < 0.05. Due to the retrospective nature of this study and the inclusion criteria, it was impossible to determine the sample size in advance. Therefore, post hoc, we performed a power analysis using G*Power program to verify the adequacy of the sample size. The effect size was determined based on previous research^23^. The calculated sample size of 36, with an effect size of 0.50 and a significance level (α error) of 0.05, yielded a statistical power of 0.92. This power value significantly exceeded the conventionally acceptable threshold of 0.80, indicating that our sample size was indeed appropriate for our research objectives.

### Ethics declarations

This study adhered to the tenets of the Declaration of Helsinki and was approved by the Institutional Review Board of Hamamatsu University School of Medicine (IRB approval no., 22-243).

### Consent to participate

The requirement for informed consent was waived due to the retrospective nature of the research.

## Results

Of the enrolled 36 patients, most were females (n = 35, 97.2%), and the number of male patients was small (n = 1, 2.8%). Therefore, the male patient was excluded from the study, and the data from 35 female patients were analysed.

The mean age, height, weight, body mass index, and disease duration were 68 ± 6.2 years, 151 ± 5.9 cm, 50 ± 7.3 kg, 22 ± 2.8 kg/m^2^, and 14 ± 9.8 years, respectively. Among the current treatments, 34 (97%), 8 (23%), 9 (26%), and 20 (57%) patients used DMARDs (including methotrexate, biologics, concomitant steroids, and concomitant nonsteroidal anti-inflammatory drugs, respectively (Table 1).

**Table 1:** Demographic characteristics, disease activity assessment, and physical function assessment data.

Sex (females)	35
Age (years)	68 ± 6.2*
Height (cm)	151 ± 5.9*
Weight (kg)	50 ± 7.3*
Body mass index (kg/m^2^)	22 ± 2.8*
Disease duration (years)	14 ± 9.8*
No. of patients using DMARDs (including MTX)	34 (97%)
No. of patients using biologics	8 (23%)
No. of patients using steroids	9 (26%)
No. of patients using NSAIDs	20 (57%)
Assessment tools in the treatment of rheumatoid arthritis	
DAS28-CRP	2.3 ± 1.0*
SDAI	9.7 ± 8.1*
CDAI	9.2 ± 7.8*
van der Heijde’s-mTSS	
Hand	137 ± 39*
Foot	60 ± 33*
Total	196 ± 69*
mHAQ-DI	0.20 ± 0.26*
Assessment tools in rehabilitation therapy	
10-Meter walk test	6.6 ± 1.1*
Timed up and go test	7.9 ± 1.4*
Functional reach test	25 ± 5.9*
Disability of the arm, shoulder, and hand (DASH)	27 ± 18*
Functional independence measure (FIM)	124 ± 0.94*

This table shows the characteristics of the subjects and shows the average values of sex, age, height, weight, BMI, and disease duration. The subjects were 35 females only. It also shows the types of therapeutic drugs and their proportion to the total. In addition, the average values of DAS28-CRP, SDAI, CDAI, van der Heijde's-mTSS (hand, foot, total), and mHAQ-DI are listed as disease activity indicators for rheumatoid arthritis. Assessment tools in rehabilitation therapy include the average values of the 10-Meter Walk Test; Timed Up and Go Test; Functional Reach Test; Disability of the Arm, Shoulder, and Hand (DASH), and Functional Independence Measure (FIM). It should be noted that all patients included in the study had all these assessments performed.

DMARDS, disease-modifying antirheumatic drugs; MTX, methotrexate; NSAIDs, nonsteroidal anti-inflammatory drugs; DAS28-CRP, Disease Assessment Score 28-C-reactive protein; SDAI, Simplified Disease Activity Index; CDAI, Clinical Disease Activity Index; mTSS, modified Total Sharp Score; mHAQ-DI, modified Health Assessment Questionnaire disability index; SD, standard deviation.

*Data represent the mean ± SD.

### Correlations between RA disease activity (DAS28-CRP, SDAI, and CDAI) and physical function (10 MWT, TUG, FRT, and DASH) assessments used in rehabilitation

**Figure 1:**
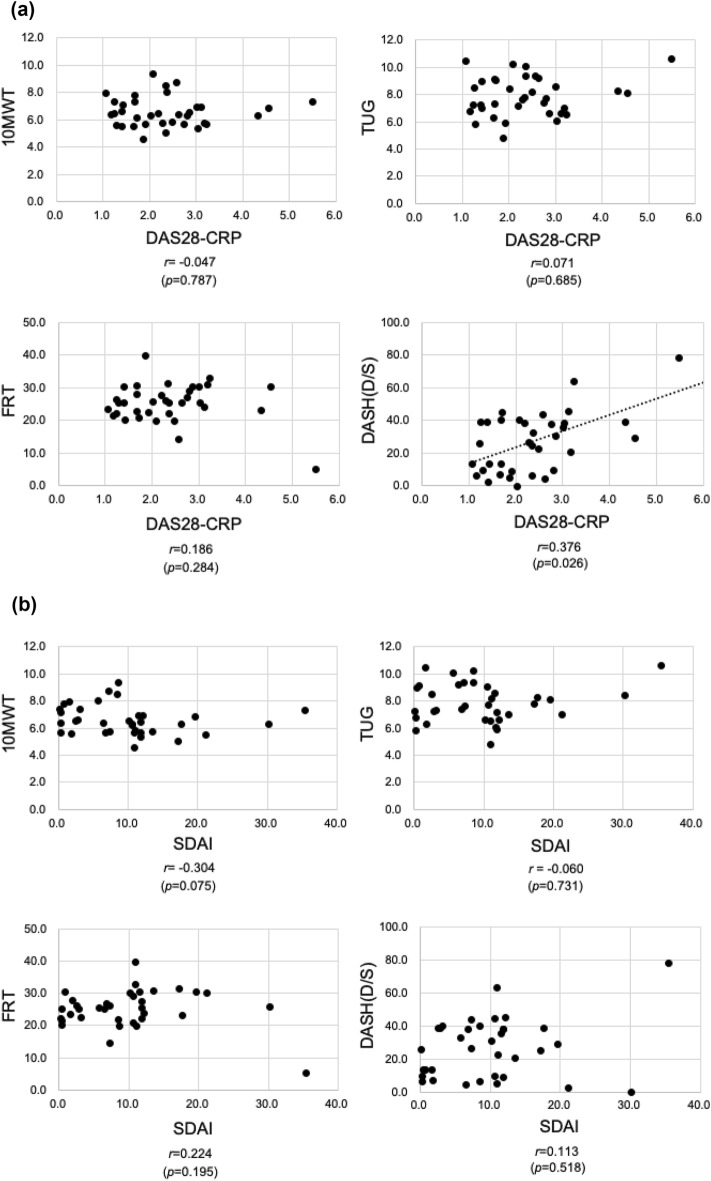

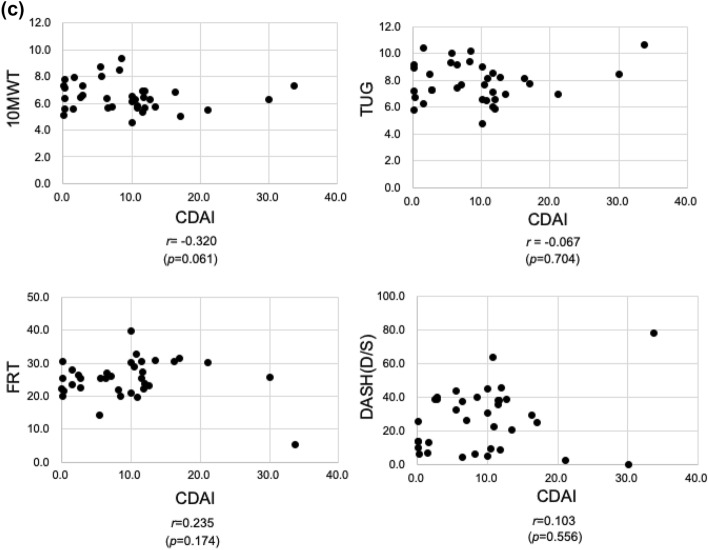
(**a**) Plots of DAS28-CRP and physical functional assessment scores for all patients. The DAS28-CRP correlated only with DASH among physical function evaluations. (**b**) Plots of SDAI and physical functional assessment scores for all patients. SDAI did not correlate with any physical function evaluations. (**c**) Plots of CDAI and physical functional assessment scores for all patients. CDAI did not correlate with any physical function evaluations. The R-values show the correlation coefficients. The dotted lines show the regression lines.

DAS28-CRP correlated with only DASH (*r* = 0.376, *p* = 0.026) among the physical function evaluations. SDAI and CDAI did not correlate with 10 MWT, TUG, FRT, or various physical function assessments such as DASH (Fig. 1).

### Roentgenographic joint destruction assessment (mTSS-hand, mTSS-foot, mTSS-total) and physical function (10 MWT, TUG, FRT, and DASH) assessments used in rehabilitation

**Figure 2:**
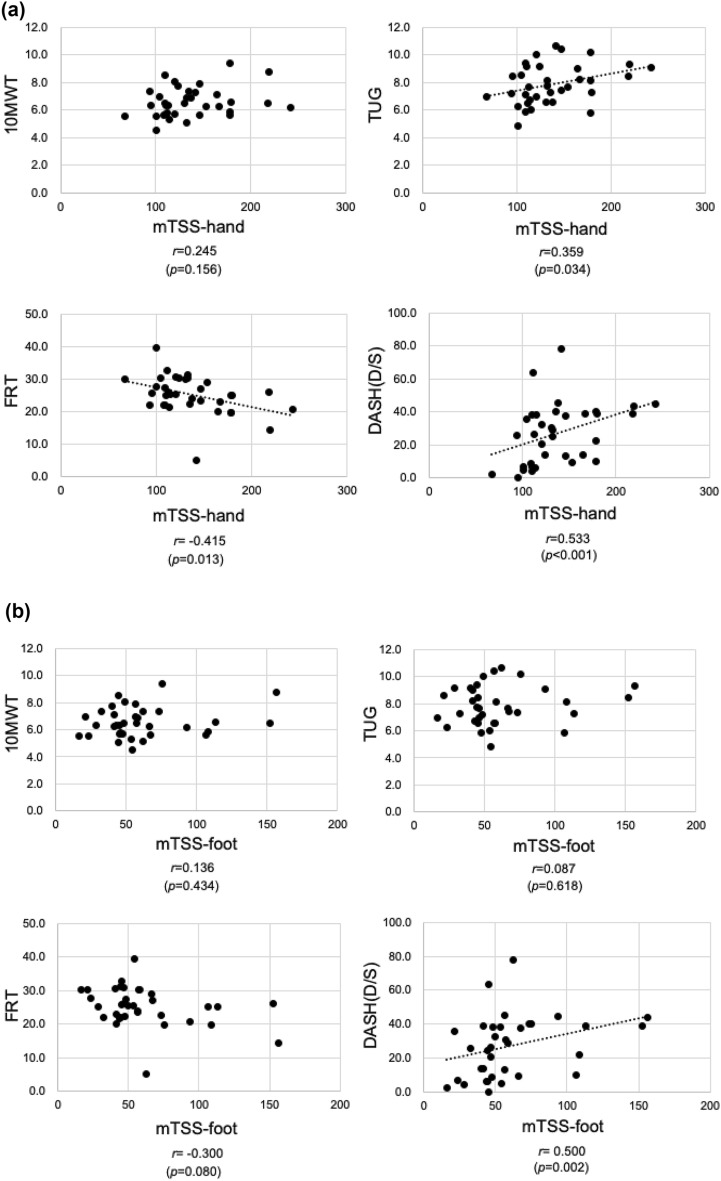

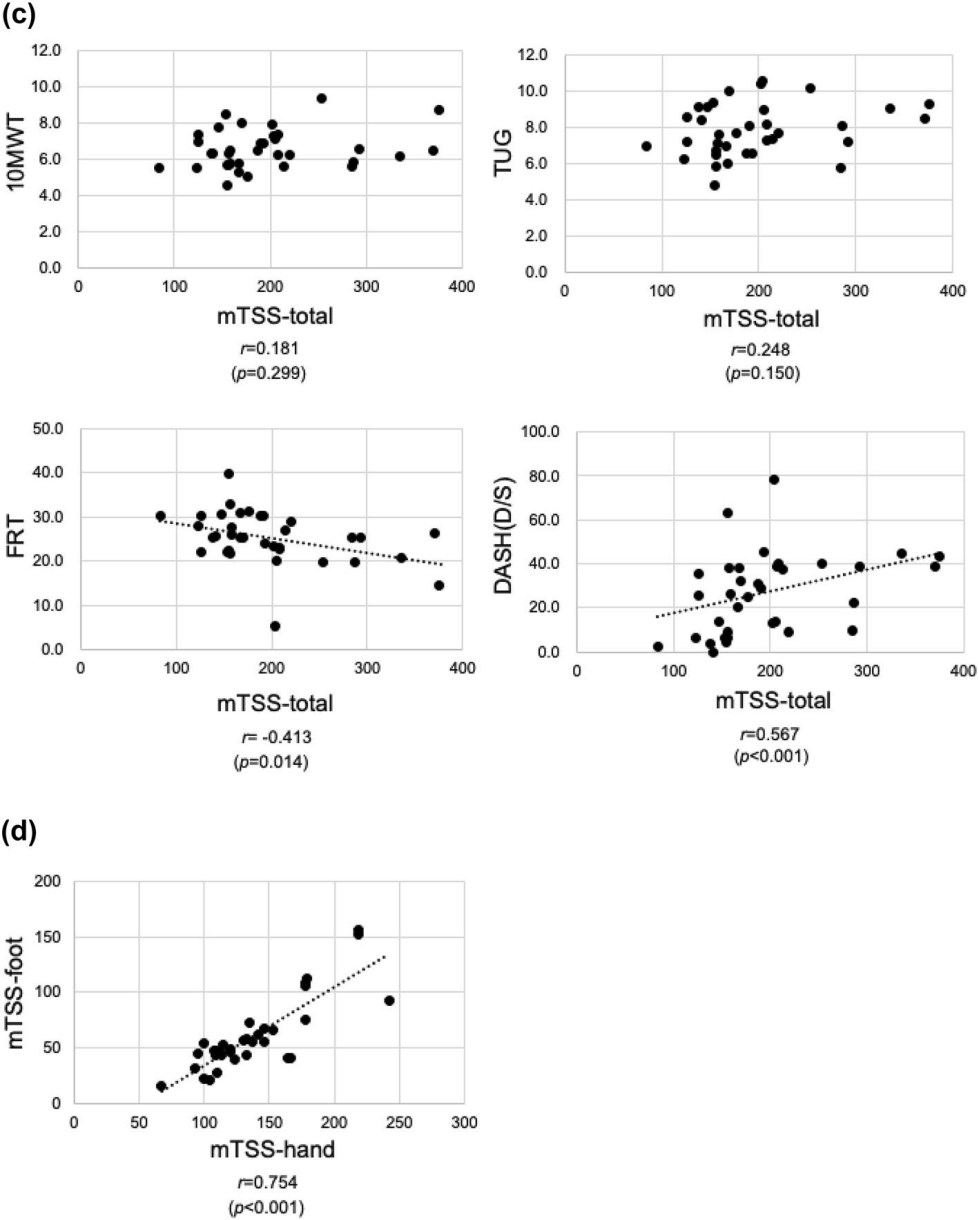
(**a**) Plots of mTSS-hand and physical functional assessment scores for all patients. The mTSS-hand correlated with TUG, FRT, and DASH among the physical function evaluations. (**b**) Plots of mTSS-foot and physical functional assessment scores for all patients. The mTSS-foot correlated only with DASH. (**c**) Plots of mTSS-total and physical functional assessment scores for all patients. The mTSS-total correlated with FRT and DASH. (**d**) Plots of mTSS-hand and mTSS-foot scores for all patients. The mTSS-hand score correlated with the mTSS-foot score. The R-values show the correlation coefficients. The dotted lines show the regression lines.

The mTSS-hand, which is roentgenographic joint destruction, correlated with TUG (*r* = 0.359, *p* = 0.034), FRT (*r* = − 0.415, *p* = 0.013), and DASH (*r* = 0.533, *p* < 0.001) among the physical function evaluations. However, only the DASH score correlated with the mTSS-foot (*r* = 0.500, *p* = 0.002). A correlation was found between mTSS-total and the FRT (*r* = − 0.413, *p* = 0.014) and DASH scores (*r* = 0.567, *p* < 0.001). In addition, the mTSS-hand and mTSS-foot scores were correlated (Fig. 2).

**Figure 3:**
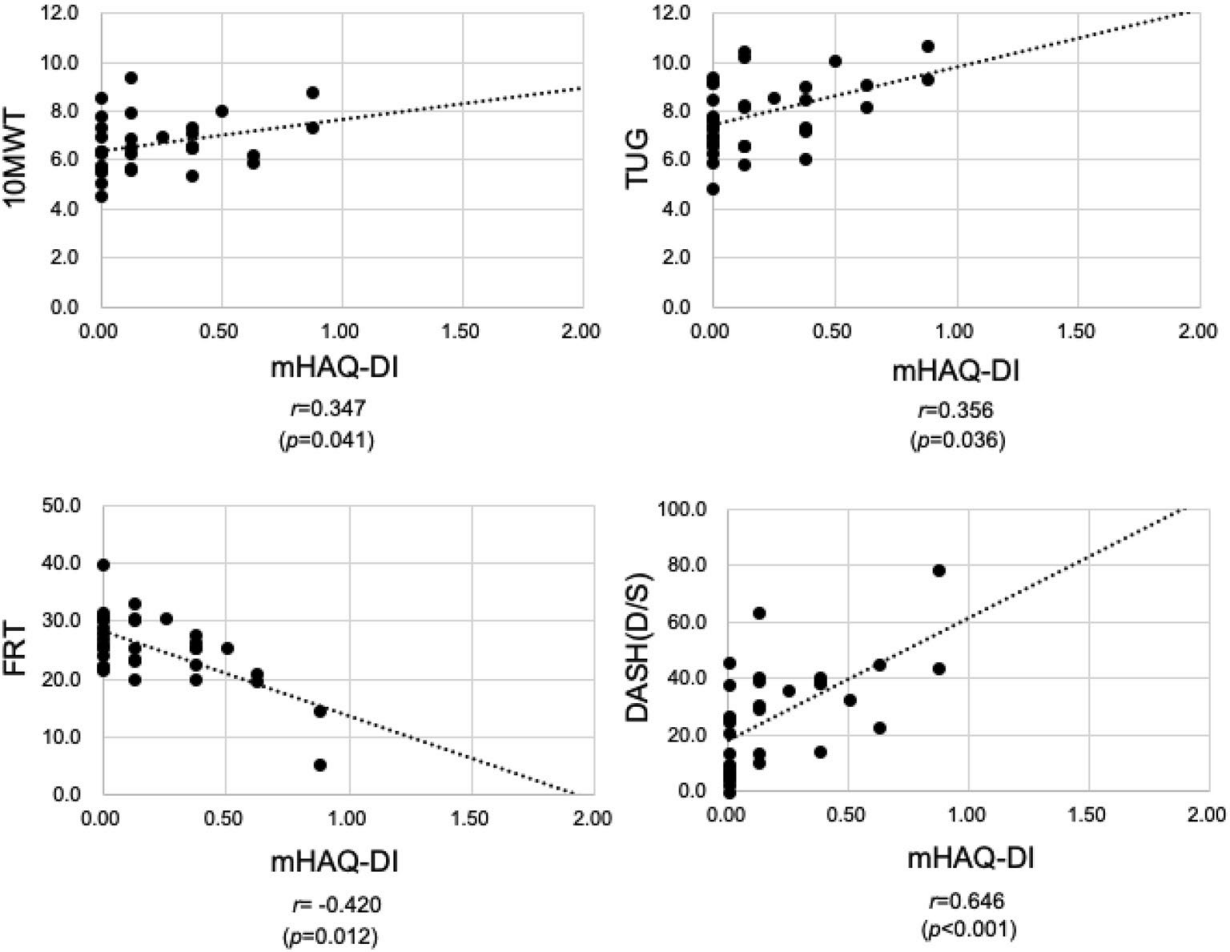
Plots of mHAQ-DI and physical functional assessment scores for all patients. The mHAQ-DI correlated with all physical function evaluations, 10 MWT, TUG, FRT, and DASH. The R-values show the correlation coefficients. The dotted lines show the regression lines.

The mHAQ correlated with all the physical function evaluations as follows: 10 MWT (*r* = 0.347, *p* = 0.041), TUG (*r* = 0.356, *p* = 0.036), FRT (*r* = − 0.420, *p* = 0.012), and DASH score (*r* = 0.646, *p* < 0.001) (Fig. 3).

### Correlation between RA disease activity (DAS28-CRP, SDAI, and CDAI), ADL (FIM), and QOL (SF-36®) assessments

**Figure 4:**
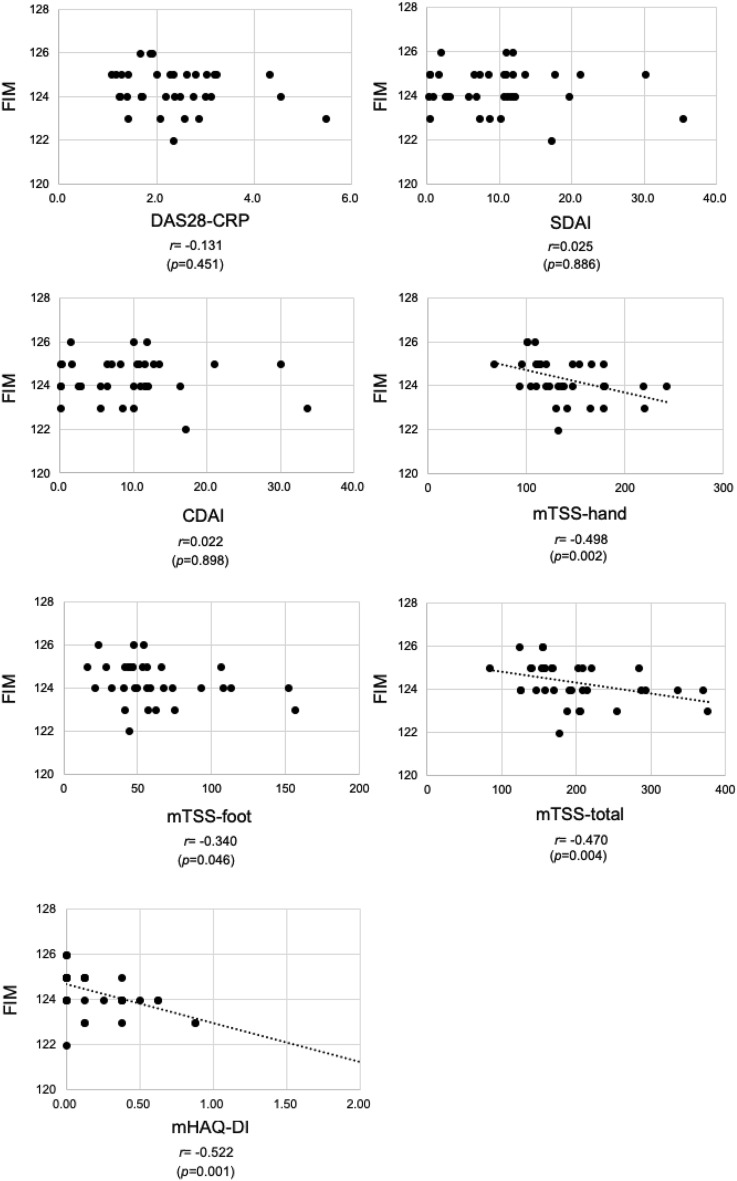
Plots of disease activity and FIM scores for all patients. The correlation between RA disease activity index and FIM was observed in mTSS-hand, mTSS-total, and mHAQ. The R-values show the correlation coefficients. The dotted lines show the regression lines.

A correlation between the RA disease activity index and FIM was observed in the mTSS-hand (*r* = − 0.498, *p* = 0.002), mTSS-total (*r* = − 0.470, *p* = 0.004), and mHAQ-DI (*r* = − 0.522, *p* = 0.001) (Fig. 4).

Correlations between the RA disease activity index and SF-36® and DAS28-CRP correlated with RF (*r* = − 0.426), RP (*r* = − 0.566), BP (*r* = − 0.366), SF (*r* = − 0.401), RE (*r* = − 0.458), and MH (*r* = − 0.413). Correlation was observed between the mTSS-hand and RP (*r* = − 0.300). The mHAQ-DI correlated with RP (*r* = − 0.411) (Table 2).

**Table 2:** Correlations between assessment tools in the treatment of rheumatoid arthritis and SF36 score.

					van der Heijde’s-mTSS			
	Mean ± SD score	DAS28-CRP	SDAI	CDAI	Hand	Foot	Total	mHAQ-DI
		r	r	r	r	r	r	r
PF	33 ± 14	− 0.426*	− 0.146	− 0.122	− 0.226	− 0.197	− 0.223	− 0.317
RP	44 ± 11	− 0.566**	− 0.221	− 0.218	− 0.300*	− 0.295	− 0.329	− 0.411*
BP	47 ± 8.5	− 0.366*	− 0.265	− 0.257	− 0.168	− 0.203	− 0.177	− 0.302
GH	45 ± 8.3	− 0.125	0.079	0.084	− 0.172	− 0.115	− 0.156	− 0.069
VT	50 ± 8.3	− 0.260	− 0.115	− 0.130	0.069	0.086	0.055	− 0.054
SF	48 ± 12	− 0.401*	− 0.015	0.002	0.060	0.143	0.094	− 0.193
RE	49 ± 9.9	− 0.458**	− 0.292	− 0.302	− 0.048	0.028	− 0.054	− 0.125
MH	51 ± 9.6	− 0.413*	− 0.201	− 0.221	− 0.022	0.025	− 0.040	− 0.019

This table shows the results of the correlation between the RA disease activity index and the eight subscales of the SF-36® that assessed QOL. Correlations between the RA disease activity index and SF-36® and DAS28-CRP correlated with PF (*r* = − 0.426), RP(*r* = − 0.566), BP(*r* = − 0.366), SF (*r* = − 0.401), RE(*r* = − 0.458), and MH(*r* = − 0.413). A correlation was observed between the mTSS-hand and RP (*r* = − 0.300). The mHAQ was correlated with RP (*r* = − 0.411).

DAS28-CRP, Disease Assessment Score 28-C-reactive protein; SDAI, Simplified Disease Activity Index; CDAI, Clinical Disease Activity Index; mTSS, modified Total Sharp Score; mHAQ-DI, modified Health Assessment Questionnaire disability index; SD, standard deviation; PF, Physical Function; RP, Role Physical; BP, Bodily Pain; GH, Global Health; VT, Vitality; SF, Social Function; RE, Role Emotional; MH, Mental Health. *p< 0.05; **p< 0.01.

## Discussion

We investigated the relationship between RA disease activity indices (DAS28-CRP, SDAI, and CDAI)/roentgenographic joint destruction assessment (mTSS)/simple ADL assessment (mHAQ) and physical function assessments (10 MWT, TUG, FRT, and DASH)/detailed ADL assessment (FIM)/QOL assessment (SF-36) used in rehabilitation therapy. The results showed that RA disease activity indices do not necessarily reflect the physical functions of female patients with RA. To the best of our knowledge, our study is the first to comprehensively examine the relationship between assessments related to RA treatment and those used in rehabilitation treatment.

We examined the relationship between RA disease activity indices (DAS28-CRP, SDAI, and CDAI) and physical function assessments (10 MWT, TUG, FRT, and DASH) used in rehabilitation therapy. DAS28-CRP correlated only with DASH, whereas SDAI and CDAI did not correlate with any physical function evaluation. Furthermore, none of the RA disease activity indicators, DAS28-CRP, SDAI, and CDAI, correlated with any of the lower extremity functional evaluations of 10 MWT, TUG, and FRT. This result indicates that RA disease activity indices do not necessarily reflect physical function, particularly lower-extremity function, in patients with RA. Even though RA disease activity indices (DAS28-CRP, SDAI, and CDAI) are widely used in RA treatment strategies, they are mainly used to evaluate the efficacy of drug therapy^5,6^. It should be noted here that the 28 specific joints assessed using DAS28-CRP, SDAI, and CDAI included the 20 fingers and two shoulders, elbows, wrists, and knees. This means that lower extremity function was under-assessed, with evaluations including only around 93% of the joints of the upper extremity, 70% of the finger joints, and 7% of the lower extremity. This could be a reason why DAS28-CRP only correlated with DASH, indicating upper extremity function, rather than with the lower extremity functions such as the 10 MWT, TUG, and FRT. Other studies have reported similar results regarding the correlation between DASH and DAS28^24^. A report on quick-DASH that highly correlated with DASH also showed a correlation with DAS28, which is also a disease activity index for RA^11,12^. Although the SDAI and CDAI were not correlated with DASH in our results, other studies have reported correlations between SDAI/CDAI and DASH/quick-DASH^11,12^. This may be due to the difference in the number of cases.

In our study, DAS28-CRP, SDAI, and CDAI did not correlate with lower extremity function assessment, but another report found a negative correlation between DAS28-CRP, CDAI, and walking speed in female patients with RA^13^. This may be due to the use of a portable triaxial accelerometer rhythmogram device, which is different from our gait assessment. Importantly, the lower extremity functional assessments we used, including 10 MWT, TUG, and FRT, have been reported to be useful in predicting fall risk and reduced mobility^25–27^. Evaluation of walking function is important to prevent falls, particularly for patients with RA who have fragile bones that are prone to fractures. Recently, the short physical performance battery (SPPB), which consists of three tests: a balance test, a walking test, and a chair-standing test, has been commonly used to detect declines in physical function^28^; moreover, it is considered to be useful for patients with RA. In our study, among the RA disease activity indices, only DAS28-CRP correlated with DASH, an assessment of upper extremity function in RA patients, but not with lower extremity function. Therefore, it is important to perform 10MWT, TUG, and FRT that could evaluate lower extremity function. If these functions are found to be impaired, it is important to perform rehabilitation treatment centred on appropriate physical therapy.

We used van der Heijde's-mTSS with radiographs of the hands and feet to assess joint destruction. The mTSS-total correlated with FRT and DASH; the mTSS-hand correlated with TUG, FRT, and DASH among physical function evaluations; and the mTSS-foot correlated only with DASH. In this study, as the mTSS-hand score increased, the DASH score also increased. A previous study using the Genant-mTSS showed a correlation between mTSS-hand and DASH scores, which is consistent with our finding^23^. There was no correlation between the mTSS-hand and the 10 MWT, which indicates walking speed. However, interestingly, the mTSS-hand was correlated with the TUG, which indicates the balance function of the lower extremities. As the mTSS-hand score increased, the number of seconds of the TUG increased. This indicates that patients with advanced hand joint destruction have poor balance function in their lower extremities. Furthermore, the mTSS-hand correlated with the FRT, which indicates the balance function of the lower limbs, and as the mTSS-hand score increased, the FRT increased. This indicates that patients with advanced hand joint destruction have poor balance function in their lower extremities. However, to the best of our knowledge, no studies have reported that the mTSS-hand is correlated with lower extremity function evaluations of TUG and FRT. The mTSS-hand is an image evaluation of the fingers, and the progression of finger joint destruction indicates the progression of RA, but interestingly, it may also reflect the degree of lower extremity function. As joint destruction due to RA progresses, physical function is expected to decline. Therefore, mTSS-hand results showing the progression of joint destruction of the hand indicate that the balance function of the lower extremity may be impaired. Furthermore, mTSS-foot was correlated with DASH, an upper extremity functional assessment, and to our knowledge, no study has reported this result. This result indicates that advanced foot joint destruction is accompanied by a decline in upper extremity function. This may be related to the fact that there is a correlation between mTSS-foot and mTSS-hand. In summary, the mTSS-total correlated with FRT and DASH; the mTSS-hand correlated with TUG, FRT, and DASH among physical function evaluations; and the mTSS-foot correlated only with DASH. Patients with progressive joint destruction as determined by roentgenographic evaluation of their hands using the mTSS hand are expected to have decreased balance function in their lower extremities. Therefore, we recommend evaluation using 10 MWT, TUG, and FRT, and if the results indicate that there is a risk of falling, rehabilitation treatment should be considered immediately.

The mHAQ correlated with all physical function assessments, including the 10 MWT, TUG, FRT, and DASH scores. In rehabilitation treatment, ADL evaluation is always performed using the FIM and Barthel Index (BI). Here, the mHAQ correlated with the FIM. There are many reports in RA treatment where HAQ and mHAQ are frequently used for ADL evaluation. The mHAQ used in this study was simplified by selecting 8 of the 20 HAQ items. A study that compared HAQ and mHAQ and examined their correlation with SF-36® and the Arthritis Impact Measurement Scales (AIMS) found that mHAQ and HAQ may be applicable as measures of physical capacity in patients with RA^9^. However, another study reported that in patients with RA treated with infliximab, the mean mHAQ score changed similarly to the HAQ-DI, but the mean HAQ-DI was significantly higher than the mean mHAQ score^29^. Here, DAS28, SDAI, and CDAI, which indicate disease activity in RA, were not correlated with FIM, suggesting that ADL cannot necessarily be assessed by evaluating arthritis status alone. In our study, we found a correlation between mHAQ and FIM, and we consider that evaluation using mHAQ is useful. Regarding the usefulness of HAQ, it is recommended in clinics when conducting clinical studies on RA, as it provides continuous clinically useful information^30^. Ideally, we recommend using FIM, including cognitive function evaluation, when performing ADL evaluation in regular outpatient RA treatment. However, since FIM is slightly complicated to measure, we consider that the simpler mHAQ could be used for evaluation in RA treatment.

The relationship between the RA disease activity index and QOL was investigated using SF-36®. The results showed that DAS28-CRP correlated with physical function, RP, BP, SF, RE, and MH among the eight domains of the SF-36®. The mTSS-hand, which is a radiographic evaluation of joint destruction, correlated with RP. Other studies also reported a negative correlation between SF-36® and DAS-28 scores^31^. This is because DAS-28 includes many findings of finger arthritis; therefore, the condition of the fingers may be involved in the patient's QOL in RA. This is also suggested by the fact that our study found a correlation between DAS-28 and DASH scores. It has been reported that the DASH score is strongly correlated with the HAQ and physical components among the SF-36® scores^24^. In our study, the mHAQ correlated only with physical function, RP, and BP. According to a study that investigated whether treatments to achieve remission in RA improve all aspects of health-related quality of life (HRQOL), the study found that remission optimizes HRQOL, but normalisation does not; the authors stated the need for treatment strategies targeting HRQOL^32^.

Here, we will further discuss upper extremity dysfunction in RA patients.　DAS28, mHAQ, mTSS-hand, and mTSS-total correlated with the DASH score, which indicates upper extremity dysfunction. A survey of the type of functions and activities in daily life affected by RA conducted through telephone interviews with 143 patients with RA showed that 87.4% of the participants had at least one functional disability resulting from RA affecting everyday life^33^. Furthermore, the most commonly mentioned disabilities were walking and opening jars. Therefore, patients with RA exhibit a very high rate of functional impairment, signifying the need and importance for evaluation of walking and finger functions. The survey also reported that older people were more likely to mention issues related to upper extremity function^33^. Our research also indicates that disease activity and progression of joint destruction lead to dysfunction in patients with RA, especially in the upper extremities.

In summary, among the RA disease activity indices, only DAS28-CRP was correlated with DASH, an assessment of upper extremity function in patients with RA, but not with lower extremity function. Furthermore, the mTSS-total correlated with FRT and DASH; the mTSS-hand correlated with TUG, FRT, and DASH among physical function evaluations; and the mTSS-foot correlated only with DASH. Therefore, patients with advanced joint destruction in finger image evaluation using the mTSS-hand may have decreased balance function of their lower extremity and may be at risk of falling, so standard lower extremity functional evaluations such as 10 MWT, TUG, and FRT are recommended. If these functions are found to be impaired, it is important to perform rehabilitation treatment centred on appropriate physical therapy.

This study has some limitations. First, among the 36 enrolled patients, 35 were females and one patient was male. Since the male patient was excluded from the study, only the data of 35 female patients were analysed. Future research should prioritize increasing the inclusion of male subjects and conducting studies with larger, more diverse datasets. Additionally, all participants were able to walk independently without walking aids, possibly introducing selection bias regarding the physical function basis. The small sample size could also impact the results. Furthermore, the medical records utilised in this study date back a decade. The decision to reference records from 2012 to 2013 was made due to data completeness during that timeframe. Measures such as 10 MWT, TUG, FRT, and DASH are often used in physical and occupational therapy during rehabilitation. However, not all of these assessments are consistently administered. Moreover, two physical therapists assessed participants' physical function, but assessments were conducted by only one of them. Although test results were not averaged, it is considered that evaluations are unlikely to significantly differ between examiners. Nevertheless, the evaluation was not blinded and was performed by a single evaluator, which may introduce potential bias. The physical function evaluation of patients with RA did not include muscle strength or joint range of motion, which may affect upper and lower limb function. Therefore, future research should explore the inclusion of these parameters for a more comprehensive evaluation.

Moving forward, it is crucial for research to investigate how differences in pharmacological and surgical treatments received by subjects affect the relationship between assessments of disease activity and physical function. These findings could guide treatment decisions for patients with RA. Although this was a cross-sectional observational study, it is anticipated that patients with RA will experience changes in physical function as the disease progresses. Therefore, future longitudinal studies are needed to elucidate the relationship between disease activity and physical function assessments. Furthermore, as a prospective intervention study, we believe it is important to investigate the relationship between physical function and disease activity evaluations when rehabilitation treatments such as physical and occupational therapies are performed. We investigated the relationship between RA disease activity indices (DAS28-CRP, SDAI, and CDAI), mTSS and mHAQ and physical functions assessments (10 MWT, TUG, FRT, and DASH) and QOL assessments used in rehabilitation therapy. DAS28-CRP correlated only with DASH, whereas SDAI and CDAI did not correlate with any physical function evaluation. RA disease activity assessments do not necessarily reflect the physical functions and QOL of patients with RA. It is important to evaluate both upper and lower limb function and QOL in patients with RA using rehabilitation-consistent methods.

## Data Availability

The datasets generated and/or analysed during the current study are not publicly available due to the fact that these data are owned by the hospital but are available from the corresponding author on reasonable request.
